# Simulated unbound structures for benchmarking of protein docking in the Dockground resource

**DOI:** 10.1186/s12859-015-0672-3

**Published:** 2015-07-31

**Authors:** Tatsiana Kirys, Anatoly M. Ruvinsky, Deepak Singla, Alexander V. Tuzikov, Petras J. Kundrotas, Ilya A. Vakser

**Affiliations:** Center for Computational Biology, The University of Kansas, Lawrence, KS 66047 USA; United Institute of Informatics Problems, National Academy of Sciences, 220012 Minsk, Belarus; Schrödinger, Inc., Cambridge, MA 02142 USA; Department of Molecular Biosciences, The University of Kansas, Lawrence, KS 66045 USA

**Keywords:** Protein interactions, Protein docking, Molecular recognition, Conformational analysis

## Abstract

**Background:**

Proteins play an important role in biological processes in living organisms. Many protein functions are based on interaction with other proteins. The structural information is important for adequate description of these interactions. Sets of protein structures determined in both bound and unbound states are essential for benchmarking of the docking procedures. However, the number of such proteins in PDB is relatively small. A radical expansion of such sets is possible if the unbound structures are computationally simulated.

**Results:**

The Dockground public resource provides data to improve our understanding of protein–protein interactions and to assist in the development of better tools for structural modeling of protein complexes, such as docking algorithms and scoring functions. A large set of simulated unbound protein structures was generated from the bound structures. The modeling protocol was based on 1 ns Langevin dynamics simulation. The simulated structures were validated on the ensemble of experimentally determined unbound and bound structures. The set is intended for large scale benchmarking of docking algorithms and scoring functions.

**Conclusions:**

A radical expansion of the unbound protein docking benchmark set was achieved by simulating the unbound structures. The simulated unbound structures were selected according to criteria from systematic comparison of experimentally determined bound and unbound structures. The set is publicly available at http://dockground.compbio.ku.edu.

## Background

Proteins play an important role in biological processes in living organisms. Many protein functions are based on interaction with other proteins. The structural information is essential for adequate description of these interactions. Protein interaction is characterized by structural and physicochemical recognition factors [1–3], and conformational changes upon binding [4]. Computational approaches to the structural modeling of protein interactions are important, given the limitations of experimental techniques [1]. A significant progress in the computational prediction of protein-protein complexes (protein-protein docking) has been reflected in the community-wide assessment [5]. The original steric complementarity-based algorithms paved the way to knowledge-based approaches [1, 3, 6, 7] including those based on similarity to existing co-crystallized complexes, low-resolution (coarse-grained) techniques, and proteome-wide applications [2].

The docking algorithms are generally based on the concept of structure complementarity, observed in experimentally determined complexes. Thus, most docking procedures perform better when the bound (co-crystallized) protein structures are used, assuring the perfect match between the structures. Such bound docking allows one to neglect the internal degrees of freedom (structural flexibility), providing for an effective search of the six-dimensional rigid-body space of the external coordinates. However, in the real-case scenario, the bound structures of the participating proteins are unknown, and one has to rely on the unbound (e.g. crystallized separately) proteins. Because of the huge number of potentially relevant internal degrees of freedom, the problem of unbound docking is far from being solved.

The rigid-body docking of unbound proteins results in structural mismatches at the putative interfaces. Thus, one approach to the unbound docking lowers the resolution of the structures, alleviating the difference between unbound and bound structures, and decreasing the structural overlap [8]. The downside of such an approach is a lesser (low-resolution) precision of the predicted structure of the complex. An alternative paradigm is to use sophisticated scoring schemes to evaluate a large number (e.g. hundreds of thousands) of high-resolution rigid-body predictions, in anticipation that it would capture the native interface containing structural mismatches (thus having high energy) [9]. Docking approaches that explicitly search the internal coordinates are being developed [10, 11]. However, their success in the unbound docking is still limited [5]. Template-based docking approaches (structure or sequence-based) generally are based on the backbone alignment (followed by the repacking of the side chains for the final prediction). Thus, in principle, they should not depend on the bound/unbound difference in the side chains conformations. However, the difference in the backbone may affect the performance of the procedure.

For the development of docking techniques applicable to the unbound proteins, it is essential to learn the experimentally determined difference between bound and unbound states. A number of proteins have structures experimentally determined in both unbound and bound states [4, 12]. In most proteins (71 % of complexes) conformational change upon binding is < 2 Å all atoms RMSD [13]. A significant number of complexes with larger RMSD have a domain shift, where conformational changes in the domains themselves are small. Still, the other cases of large RMSD involve interface loops, which change conformation significantly upon binding. Thus, our ability to adequately address conformational changes in docking is important.

The utility of the unbound docking approaches is tested in the CAPRI blind experiment [5]. To provide consistent sets for validation of docking and scoring procedures, benchmark sets of protein-protein complexes were compiled [13, 14]. However, the number of known representative protein pairs with experimentally determined structures in both bound and unbound states is relatively small (e.g., 176 in the Weng’s Benchmark 4.0 [14]). At the same time, the number of co-crystallized complexes is much larger. A key feature in the Dockground resource [15] is flexibility, which allows users to build the datasets according to their own requirements. Such datasets can involve thousands of complexes and thus can be used for truly large-scale benchmarking of docking methodologies. Simulating the unbound structure from the bound one provides such an opportunity. Our earlier set of simulated unbound structures [13], based on an older version of Dockground, was generated by changing the side chain conformations according to the rotamer library [16]. In the current paper we describe a much larger set obtained by Langevin dynamics simulation and based on a systematic analysis of the experimentally determined bound/unbound structural differences. The set is a valuable resource for benchmarking docking procedures and development of docking methodologies.

## Methods

Protein complexes were selected from the Bound part of Dockground with the following criteria: mean area buried ≥ 500 Å^2^, include alternative binding modes, homo/hetero *n*-mers, and oligomers, and the redundancy cutoff 97 %. The resulting set contained 1918 protein-protein complexes. Program Profix from the Jackal package (http://wiki.c2b2.columbia.edu/honiglab_public/index.php/Software) was used to build the disordered residues and missing atoms.

It was expected that dynamic simulation of separate bound protein structures, without the interacting partner, would relax interface side-chain conformations constrained by the interacting partner and thus approximate the unbound form of the protein. To speed up the calculations, we chose the Langevin Dynamics (LD) simulation in CHARMM (CHARMM22 force field), with electrostatics by Generalized Born approximation, performed on each bound structure without its interacting partner. Prior to LD simulation, the initial structures from PDB files were minimized (by 50 steps of steepest descent minimization followed by 500 steps of adopted basis Newton–Raphson minimization).

In the simulation, the backbone atoms of helices and strands were constrained with a force constant of 5 kcal/mol, the temperature was set to 309.6 K, the bond lengths were fixed using shake with tolerance 1.0E-8, the friction force fbeta was 5.0, and the time of simulation was 1 ns, with 100 snapshots saved.

Protein structures that crashed during the simulation were removed. The simulation yielded 3205 protein structures. The number of resulting complexes with both proteins simulated was 1530. In 145 complexes only one partner was simulated. The structure with the largest all atoms RMSD from the bound structure was designated as the *simulated unbound structure*. Among the simulated proteins there were 1245 non-obligate and 1960 obligate complexes, according to NOXClass procedure [17].

### Comparison with experimentally determined structures

**Fig. 1:**
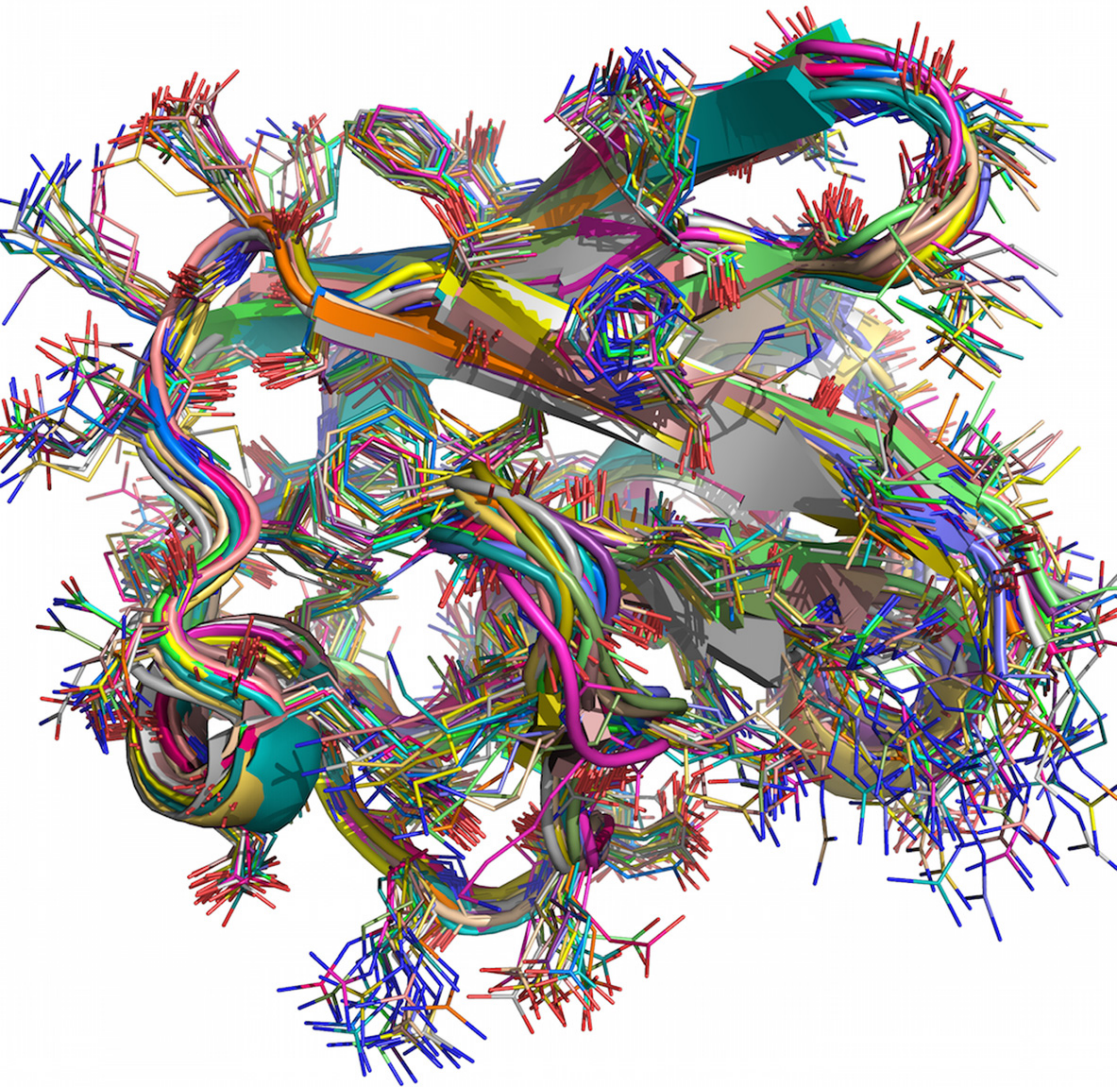
The ensemble of unbound structures of ubiquitin

Proteins in solution are dynamic and exist in an ensemble of conformations. The crystallized structure represents just one of the conformers. Our previous study showed that different crystallized conformations of a protein vary in 0 – 7 Å C^α^ RMSD interval (more in all atoms RMSD; see Fig. 1 for illustration) [12]. Recent studies also showed that molecular simulations on nanosecond time scale agree with the experimental observations of protein dynamics in solution [18–20]. Thus a valid comparison of the simulated structures with experiment should be *not with a unique X-ray structure* but with *the ensemble* of experimentally determined structures.

### Generation of the set

To validate the simulated unbound structures, an ensemble of unbound and bound experimentally determined structures from PDB was selected for six proteins: ovomucoid, pancreatic trypsin inhibitor, chemotaxis protein CheY, ubiquitin, RNase A, and lysozyme C (for details see [12]). The extent of bound to unbound change and similarity between bound and unbound ensembles was calculated in terms of *all atoms full structure* and *all atoms interface* RMSD. Interface residues were defined as those losing >1 Å^2^ of their surface upon binding.

The number of unbound structures in the ensemble ranged from 27 to 394 per protein. The difference between bound and unbound structures varied in 0.7 - 7.3 Å full structure RMSD, and in 0.3 – 11.7 Å interface RMSD. The mean RMSD between bound and unbound structures was 1.9 Å (both all atoms and interface).

**Fig. 2:**
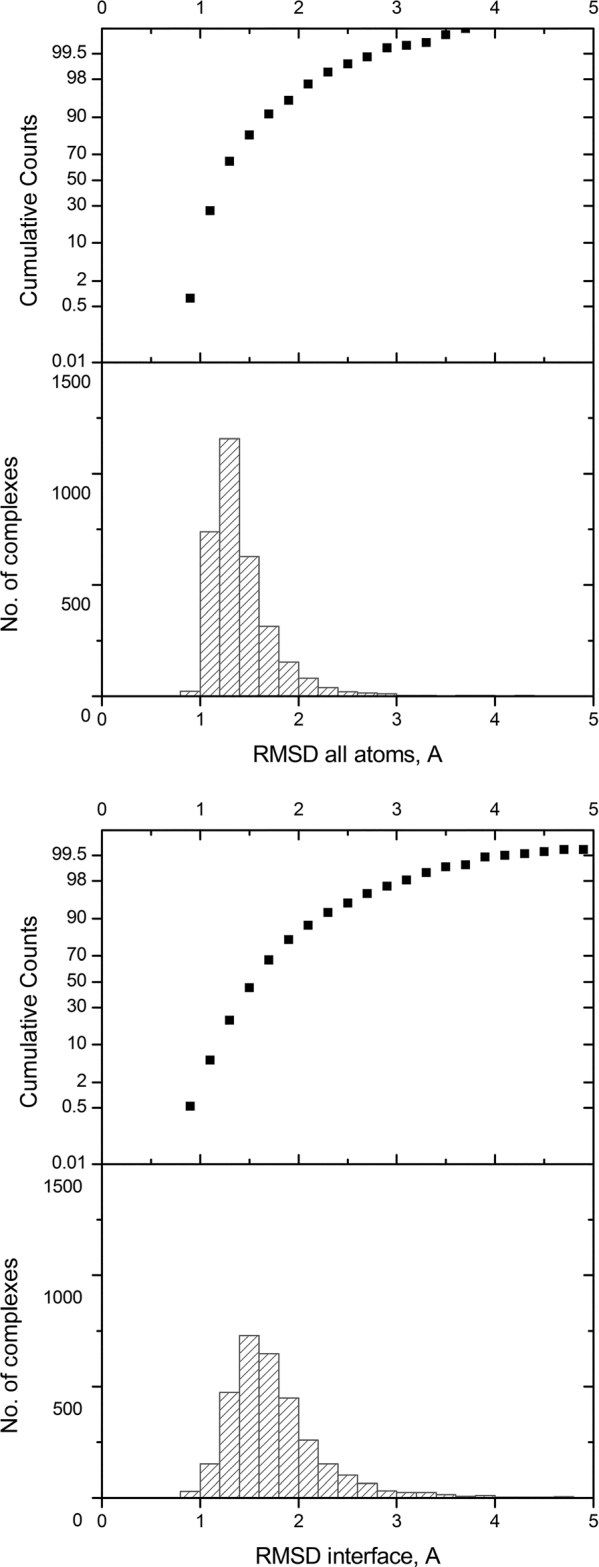
Distribution of complexes according to RMSD between bound and simulated unbound structures

The majority of simulated proteins had RMSD < 2 Å from the initial structure (Fig. 2). The average RMSD for full structure was 1.4 Å, and for the interface RMSD, 1.8 Å. The average RMSD of the simulated proteins from non-obligate and obligate complexes was very similar: 1.5 Å non-obligate, 1.4 Å obligate, for full structure RMSD, and 1.9 Å non-obligate, 1.7 Å obligate, for interface RMSD. As expected, the greater differences between the initial and the simulated structures were in the loops. A higher average interface RMSD compared to full structure RMSD is likely due to the rigidity of the protein core. The difference, albeit small, between interface RMSD of proteins from non-obligate and obligate complexes can be explained by the fact that the obligate complexes are typically bigger and contain more rigid secondary structure elements, and to a lesser extend flexible loops.

For consistency, as an option for users who would like to utilize structures of same origin, we simulated the unbound structures in cases where the X-ray unbound structure is known. The Dockground selection of monomers, with sequence identity between bound and unbound structures ≥ 97 %, and no ligands at the unbound interfaces, yielded 172 unbound/bound proteins. Among them, a single unbound structure was available for 79 proteins, with the others having multiple unbound structures. The average RMSD between bound and unbound structures was 1.2 Å (0.3 – 3.9 Å range) for full structure, and 1.5 Å (0.3 – 5.0 Å range) for the interface. The relatively small RMSD between bound and unbound structures could partially be explained by the fact that some proteins designated as monomers (and thus treated as unbound) are crystallized as homodimers. If proteins with bound/unbound RMSD ≤ 1 Å (likely not true unbound cases) are deleted, the average RMSD is 1.4 Å for the full structure, and 1.8 Å for the interface, similar to the difference between the bound and the simulated unbound structures.

**Fig. 3:**
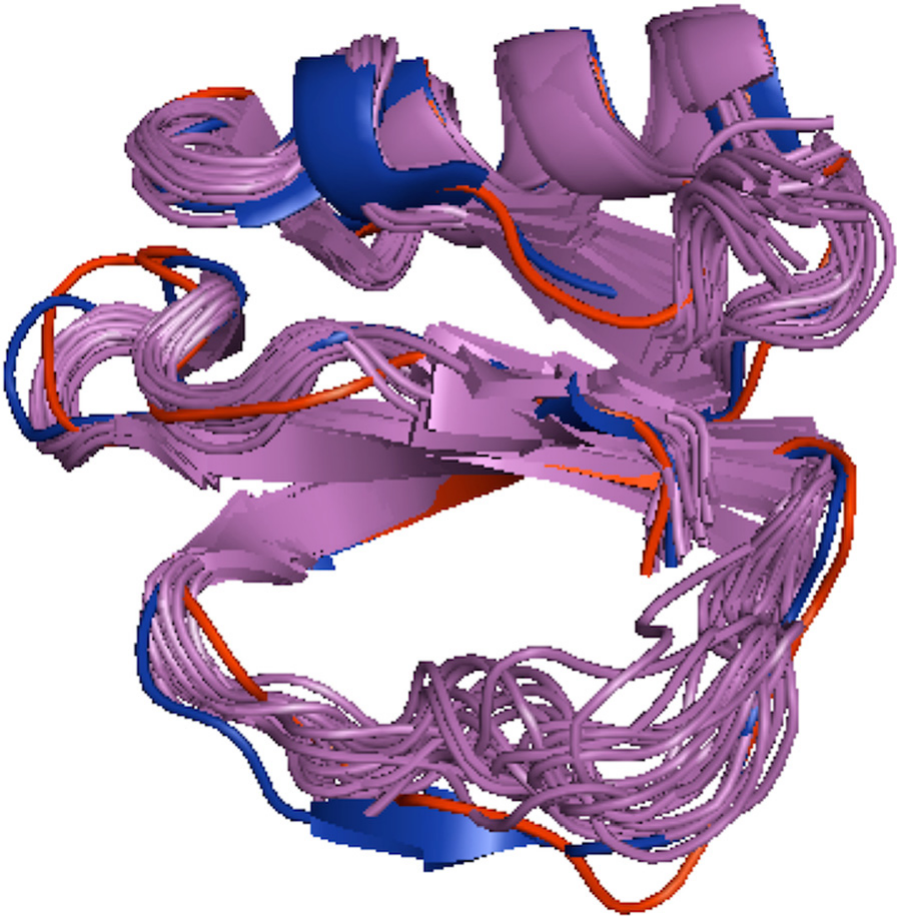
Unbound and bound structures of subtilisin-chymotrypsin inhibitor. The bound structure 1lw6 is in blue, the unbound ensemble of NMR structures 3ci2 in magenta, and the simulated unbound structure, based on 1lw6, is in red. The extended binding loop flexibility is modeled in the simulated structure

**Fig. 4:**
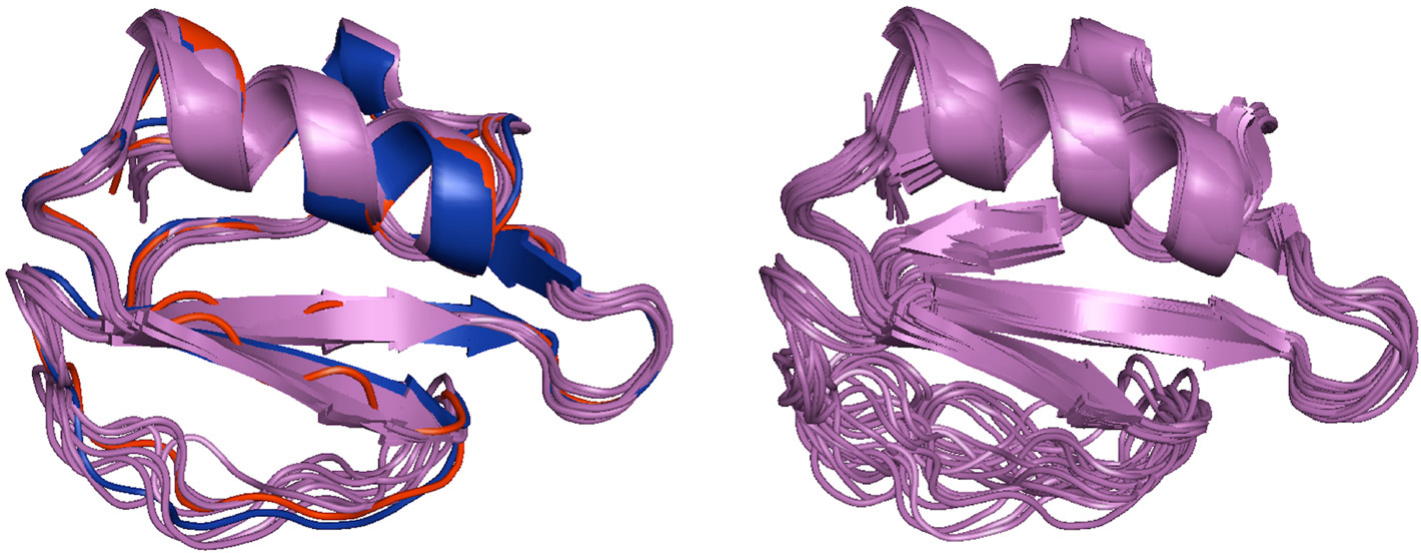
Unbound and bound structures of eglin. The bound structure 1mee is in blue, the unbound ensemble of NMR structures 1egl in magenta, and the simulated unbound structure, based on 1mee, is in red. On the left it shows selected unbound structures from NMR ensemble close to either bound or simulated structures. On the right it shows the full NMR ensemble. Some loop conformations in the unbound NMR ensemble are close to the bound conformation, whereas other conformations are similar to the simulated unbound structure. While supporting the conformational selection mechanism upon binding [12], it also confirms the validity of the simulated protocol

**Fig. 5:**
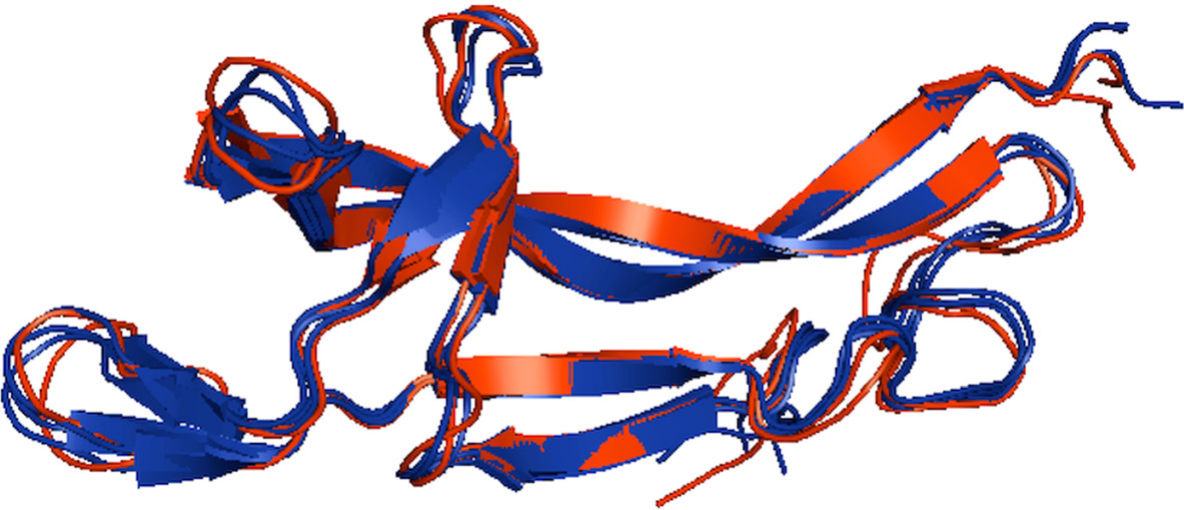
Unbound and bound structures of beta nerve growth factor from an obligate complex. The bound structure 1btg is in blue, and the simulated unbound structure is in red. Loops flexibility, important for nerve growth factor function, is modeled in simulated unbound structures. From the homodimeric complex, two crystallographically determined bound and two simulated unbound structures are shown

Examples of conformational change between the X-ray bound, unbound and simulated unbound states of proteins are shown in Figs. 3, 4, 5. Subtilisin-chymotrypsin inhibitor is known to have an extended flexible binding loop Gly54-Ile63 [21]. Its flexibility was modeled in our simulated structure (Fig. 3).

Eglin C also belongs to the potato chymotrypsin inhibitor family and has a flexible binding loop [22]. Comparison of the loop conformations of bound, unbound, and modeled structures (Fig. 4) shows that some loop conformations in the unbound NMR ensemble are close to the bound conformation, whereas other conformations are similar to the simulated unbound structure. While supporting the conformational selection mechanism upon binding [12] for eglin C suggested by the molecular simulations of serine protease inhibitor in [23], it also confirms the validity of the simulated protocol.

To further expand the pool of structures with similar bound/unbound differences (see above), obligate complexes were included as an option. Although they would not have an unbound structure *in vivo/vitro*, the algorithms that distinguish between obligate and non-obligate complexes have limited reliability [24]. The option to exclude such complexes is implemented in the user interface in the Dockground resource. An example of such complex is the nerve growth factor protein (Fig. 5), which has a conformational change upon simulation confirmed by the experimental evidence. This protein has structural flexibility in the loop regions, reflected in our simulation, and this structural malleability might be important in binding [25].

### Availability of the set

The resulting set of 3184 PDB-formatted files is available on the Dockground site (http://dockground.compbio.ku.edu) on the “Unbound - > Build Database” page, and as a “Quick Download” link. Users can download either the entire set or any combination of the available subsets. In addition to the obligate and/or non-obligate complexes, the interface offers to download structures, for which simulated unbound structures were generated for both monomers in the complex or only for one. Users can also include simulated unbound structures, for which corresponding X-ray unbound structure exists in the Dockground unbound docking benchmark 3.0. The names of the files start with the PDB code of the initial bound structure, followed by _u1 or _u2 for the first and second chain in the initial complex, respectively. Chain IDs and residue numbering were kept as in the original PDB files.

## Conclusions

The Dockground public resource provides data to improve our understanding of protein–protein interactions and to assist in development of docking algorithms and scoring functions. Sets of protein structures determined in both bound and unbound states are essential for benchmarking docking procedures. However, the number of such proteins in PDB is relatively small. A radical expansion of such sets is possible if the unbound structures are computationally simulated. Such simulated unbound protein set was generated for the Dockground resource. The modeling protocol was based on 1 ns Langevin dynamics simulation. Simulated unbound structure was selected according to criteria from systematic comparison of experimentally determined bound and unbound structures. The set is publicly available at http://dockground.compbio.ku.edu.
